# Clinical relevance of somatic mutations in main driver genes detected in gastric cancer patients by next-generation DNA sequencing

**DOI:** 10.1038/s41598-020-57544-3

**Published:** 2020-01-16

**Authors:** Marina V. Nemtsova, Alexey I. Kalinkin, Ekaterina B. Kuznetsova, Irina V. Bure, Ekaterina A. Alekseeva, Igor I. Bykov, Tatiana V. Khorobrykh, Dmitry S. Mikhaylenko, Alexander S. Tanas, Sergey I. Kutsev, Dmitry V. Zaletaev, Vladimir V. Strelnikov

**Affiliations:** 1 Medical Genetics Laboratory, I.M. Sechenov First Moscow State Medical University, Moscow, 119991 Russian Federation; 2 Epigenetics Laboratory, Research Centre for Medical Genetics, Moscow, 115522 Russian Federation; 3 Department No. 1, Medical Faculty, Faculty Surgery, I.M. Sechenov First Moscow State Medical University, Moscow, 119991 Russian Federation; 4 N.A. Lopatkin Research Institute of Urology and Interventional Radiology – branch of the National Medical Research Radiologiсal Center, Moscow, 105425 Russian Federation

**Keywords:** Gastric cancer, Prognostic markers

## Abstract

Somatic mutation profiling in gastric cancer (GC) enables main driver mutations to be identified and their clinical and prognostic value to be evaluated. We investigated 77 tumour samples of GC by next-generation sequencing (NGS) with the Ion AmpliSeq Hotspot Panel v2 and a custom panel covering six hereditary gastric cancer predisposition genes (*BMPR1A, SMAD4, CDH1, TP53, STK11* and *PTEN*). Overall, 47 somatic mutations in 14 genes were detected; 22 of these mutations were novel. Mutations were detected most frequently in the *CDH1* (13/47) and *TP53* (12/47) genes. As expected, somatic *CDH1* mutations were positively correlated with distant metastases (p = 0.019) and tumours with signet ring cells (p = 0.043). These findings confirm the association of the *CDH1* mutations with diffuse GC type. *TP53* mutations were found to be significantly associated with a decrease in overall survival in patients with Lauren diffuse-type tumours (p = 0.0085), T3-T4 tumours (p = 0.037), and stage III-IV tumours (p = 0.013). Our results confirm that the detection of mutations in the main driver genes may have a significant prognostic value for GC patients and provide an independent GC-related set of clinical and molecular genetic data.

## Introduction

Gastric cancer (GC) is the third leading cause of cancer-related deaths worldwide after lung cancer and breast cancer. The incidence of GC is particularly high in East Asia, including China, Japan and Korea, and in South America^1^. Based on the Lauren classification, GC is divided into two main types, namely, intestinal and diffuse, which have different epidemiological, morphological and clinical features. Intestinal GC commonly appears in elderly patients with multifocal atrophic gastritis, which is accompanied by intestinal metaplasia or dysplasia. Diffuse GC is more common in younger patients, and its association with atrophic gastritis or intestinal metaplasia is not obvious. Clinical differences between these two types reflect different mechanisms of the development and molecular pathogenesis of tumours^2^. However, Lauren’s classification is not closely associated with treatment and prognosis, necessitating the development of a classification combining clinical, morphological, and molecular features of GC in response to certain therapeutic modalities.

Comprehensive studies, including analyses of the genome, epigenome, proteome and transcriptome, offered an entirely different view on the tumour, moving it out of a single plane and into a multidimensional spatial image. The ability to determine the tumour-specific spectrum of genetic and epigenetic changes enables us to expand our understanding of the molecular pathogenesis of the tumour and to obtain information about the potential of targeted therapies. Mutational profiling is one way to classify tumours depending on the mutation spectrum into specific molecular subtypes that differ from the standard morphological classification. The results of recent studies, such as TCGA (The Cancer Genome Atlas) and ACRG (Asian Cancer Research Group), indicate that GC could be divided into four molecular subtypes with different mechanisms of pathogenesis. These subtypes are not completely consistent with the standard morphological classification and the Lauren classification^3,4^.

As whole-genome research methods are difficult to introduce into clinical practice, it is necessary to provide a reduced set of the most informative diagnostic and prognostic clinical markers. Furthermore, validation of the molecular subtypes of GC in large patient groups with different ethnic and racial backgrounds is clearly required. At present, it is already clear that there can be no universal classification for GC, and national genetic and epigenetic features should be considered.

A number of genes have been identified as driver genes in gastric cancer. However, the association between somatic mutations and clinical features has not been thoroughly elucidated to date. It is therefore important to profile the somatic mutation patterns of driver genes and potential driver genes in gastric cancer. Research investigating the somatic mutation profiles of cancer-related genes reveals the main driver mutations that determine the clinical behaviour of a tumour, its aggressiveness, invasion and metastasis, and the direction of targeted antitumour therapy. It was determined that GC is not enriched with known driver mutations. Therefore, the targeted drugs that are useful in the treatment of other tumours are not effective in GC therapy, and despite the development of novel drugs for GC, trastuzumab and ramucirumab (targeting HER2 and VEGFR2, respectively) are the only targeted therapies approved to date^5^.

Germline mutations in some driver genes determine predisposition to the development of hereditary gastric cancer. Mutations in *CDH1* are responsible for the development of early hereditary diffuse GC, as are mutations in *TP53* (Li-Fraumeni syndrome), *STK11* (Peutz–Jeghers syndrome), *SMAD4* or *BMPR1A* (gastrointestinal polyposis) and *PTEN* (Cowden syndrome)^6^. Thus, it is advisable to combine the *BMPR1A, SMAD4, CDH1, TP53, STK11* and *PTEN* genes into a targeted sequencing panel that will provide significant information on the mutational profile of gastric tumours in both hereditary and sporadic cancer, which can be associated with the clinical and pathomorphological features of the disease. Screening for mutations in these genes might be important to determine germline mutations in patients with both early manifestation and/or family history, as well as for somatic profiling of sporadic GC.

Research examining the main driver mutations in the tumour is critical for accurate personalized medicine. A specific profile of somatic mutations and their combinations may indicate more aggressive behaviour, invasion and metastasis and may represent a diagnostic or prognostic marker.

To investigate the GC mutation profile and determine its prognostic value, we conducted a study of 77 GC tumour samples using next-generation sequencing (NGS) on both the Ion AmpliSeq Cancer Hotspot Panel v2, covering mutation hotspots in 50 cancer-related genes, and a custom panel covering six hereditary gastric cancer predisposition genes (*BMPR1A, SMAD4, CDH1, TP53, STK11* and *PTEN*).

## Results

### Spectrum of identified somatic mutations

NGS analysis of tumour samples from 77 gastric cancer patients revealed 47 somatic mutations in 14 of the 51 genes initially selected for this study, either because the genes harboured the oncogenic mutational “hot spots” or because they were associated with the development of hereditary GC. In this paper, we report as mutations only the genetic variants that are either notably rare or absent in populations (assessed with gnomAD) or have previously been classified as pathogenic/likely pathogenic in other studies. Genetic variants with MAF > 0.0001 were excluded from the analysis. For genetic variants not previously reported in human mutation databases, we performed *in silico* pathogenicity estimation (see below).

DNA sequencing with our hereditary GC panel revealed a total of 36 mutations (Table 1), with the most frequently mutated genes being *CDH1* (13/36) and *TP53* (12/36), and the other genes being distributed as follows: *SMAD4* (6/36), *STK11* (3/36), *PTEN* (1/36), and *BMPR1A* (1/36). One of the *TP53* gene mutations, NM_000546.5:c.743 G > A:p. R248Q, was detected in two cases, which is in line with TCGA data, where this mutation is also recurrent.

**Table 1:** Mutations detected in 77 gastric tumours by a custom HGC panel addressing hereditary cancer syndromes.

Gene	Genetic variant	rsID	Variant position according to hg19	Pathogenicity (ClinVar)	MAF (gnomAD)	# of cases
*TP53*	NM_000546.5: c.257_279del: p.Arg86fs	—	chr17:7579408- 7579430	—	—	1
*TP53*	NM_000546.5: c. 517 G > T: p.V173L	—	chr17:7578413	—	—	1
*TP53*	NM_000546.5: c.892 G > A: p.E298K	rs201744589	chr17:7577046	Uncertain significance	A = 0.000012	1
*TP53*	NM_000546.5: c.742 C > T: p.R248W	rs121912651	chr17:7577539	Pathogenic	T = 0.000004	1
*TP53*	NM_000546.5: c.473 G > A: p.R158H	rs587782144	chr17:7578457	Pathogenic/Likely pathogenic	A = 0.000004	1
*TP53*	NM_000546.5: c. 695 T > C: p.I232T	rs587781589	chr17:7577586	Likely pathogenic	—	1
*TP53*	NM_000546.5: c.536 A > G: p.H179R	—	chr17:7578394	Likely pathogenic	G = 0.000004	1
*TP53*	NM_000546.5: c. 734 G > A p.G245D	rs121912656	chr17:7577547	—	A = 0.000004	1
*TP53*	NM_000546.5: c.395 A > G: p.K132R	rs1057519996	chr17:7578535	Likely pathogenic	G = 0.00000	1
*TP53*	NM_000546.5: c.524 G > A: p.R175H	rs28934578	chr17:7578406	Pathogenic	A = 0.000004	1
*TP53*	NM_000546.5: c.743 G > A p.R248Q	rs11540652	chr17:7577538	Pathogenic/Likely pathogenic	A = 0.000020	2
*TP53*	NM_000546.5: c.193 A > T: p.R65*	—	chr17:7579494	—	—	1
*CDH1*	NM_004360.4: с.907 A > C: pT303P	—	chr16:68845661	—	—	1
*CDH1*	NM_004360.4: c.1198 G > A: p.D400N	—	chr16:68847276	—	—	1
*CDH1*	NM_004360.4: c.1199 A > T: p.D400V	—	chr16:68847277	—	—	1
*CDH1*	NM_004360.4: c.531 + 2_15del	—	chr16:68842472- 68842485	—	—	1
*CDH1*	NM_004360.4: c.641 T > C: p.L214P	—	chr16:68842705	—	—	1
*CDH1*	NM_004360.4: c.641 T > A: p.L214Q	—	chr16:68842705	—	—	1
*CDH1*	NM_004360.4: c.2512 A > G: p.S838G	rs121964872	chr16:68867265	Conflicting interpretations of pathogenicity	G = 0.000041	1
*CDH1*	NM_004360.4: c.1320 + 2 T > G	—	chr16:68847400	—	—	1
*CDH1*	NM_004360.4: c.546 A > C: p.K182N	rs201141645	chr16:68842610	Uncertain significance	C = 0.000081	1
*CDH1*	NM_004360.4: c.G638A: p.W213*	—	chr16:68842702	—	—	1
*CDH1*	NM_004360.4: c.418 C > T: p.L140F	rs758277885	chr16:68842357	—	T = 0.000004	1
*CDH1*	NM_004360.4: c.1226 G > A: p.W409*	—	chr16:68847304	—	—	1
*CDH1*	NM_004360.4: c.779 C > G: p.P260R	—	chr16:68844191	—	—	1
*BMPR1A*	NM_004329: c.250 G > A: p.A84T	—	chr10:88651903	—	—	1
*PTEN*	NM_000314.4: c.800delA: p.K267fs	rs121913289	chr10:89717775	Pathogenic	—	1
*SMAD4*	NM_005359.5: c.1157 G > A: p.G386D	rs121912580	chr18:48593406	Pathogenic	—	1
*SMAD4*	NM_005359.5: c.473 T > C: p.V158A	—	chr18:48581169	—	—	1
*SMAD4*	NM_005359.5: c. 1333 C > T: p.R445*	rs377767360	chr18:48603032	Pathogenic	T = 0.000004	1
*SMAD4*	NM_005359: c.935 C > T: p.P312L	—	chr18:48586266	—	—	1
*SMAD4*	NM_005359: c.1082 G > A: p.R361H	rs377767347	chr18:48591919	Pathogenic	—	1
*SMAD4*	NM_005359: c.1066 C > T: p.P356S	—	chr18:48591903	—	—	1
*STK11*	NM_000455.4: c.848_852del: p.S283fs	—	chr19:1221325	—	—	1
*STK11*	NM_000455.4: c.866 T > A: p.M289K	—	chr19:1221951	—	—	1
*STK11*	NM_000455: c.928 C > T: p.R310W	rs750366043	chr19:1222991	—	T = 0.000006	1

*CDH1* mutations were found in 11 patients. Although none of the *CDH1* mutations was recurrent in our study, some were found in the same codons and had similar *in silico* pathogenicity predictions. These mutations include c.641 T > C and c.641 T > A, which cause leucine 214 substitution to proline and glutamine, respectively, and are both predicted to alter protein function, or c.1199 A > T and c.1198 G > A, which change aspartic acid 400 to valine and asparagine, respectively, and apparently lead to loss of protein function and alterations in posttranslational modifications.

Upon sequencing with the CHPv2 panel, 11 mutations in 8 genes were detected: *PIK3CA* (2/11), *RB1* (3/11), and one mutation each in *CDKN2A, SMO, KRAS, EGFR, KIT*, and *KDR*. The results are presented in Table 2.

**Table 2:** Mutations detected in 77 gastric tumours by Ion AmpliSeq Cancer Hotspot Panel v2.

Gene	Genetic variant	rsID	Variant position according to hg19	Pathogenicity (ClinVar)	MAF (gnomAD)	Number of cases
*PIK3CA*	NM_006218.3: c.1633G > A: p.E545K	rs104886003	chr3:178936091	Pathogenic	A = 0.000004	1
*PIK3CA*	NM_006218.3: c.3140 A > G: p.H1047R	rs121913279	chr3:178952085	Pathogenic	G = 0.000004	1
*EGFR*	NM_005228.3: c.874 G > A: p.V292M	rs150549265	chr7:55221830	—	A = 0.000004	1
*KIT*	NM_000222.2: c.G148T: p.V50L	rs200950545	chr4:55561758	Uncertain significance	T = 0.000033	1
*KDR*	NM_002253: c.G2678C: p.G893A	—	chr4:55962446	—	—	1
*RB1*	NM_000321.2: c.2056 C > A: p.H686N	—	chr13:49033919	—	—	1
*RB1*	NM_000321.2: c.2002C > T: p.R668C	rs369755801	chr13:49033865	—	T = 0.000028	1
*RB1*	NM_000321.2: c.1690C > T: p.L564F	—	chr13:48955574	—	—	1
*CDKN2A*	NM_000077.4: c.307 C > T: p.R103W	rs767642535	chr9:21971051	Uncertain significance	A = 0.000004	1
*SMO*	NM_005631.4: c.618 G > A: p.W206*	rs751636409	chr7:128845124	—	A = 0.000008	1
*KRAS*	NM_0.004985.4: c.35 G > A: p.G12D	rs121913529	chr12:25398284	Pathogenic	A = 0.000004	1

### Clinical relevance of gastric cancer somatic mutational status

**Figure 1:**
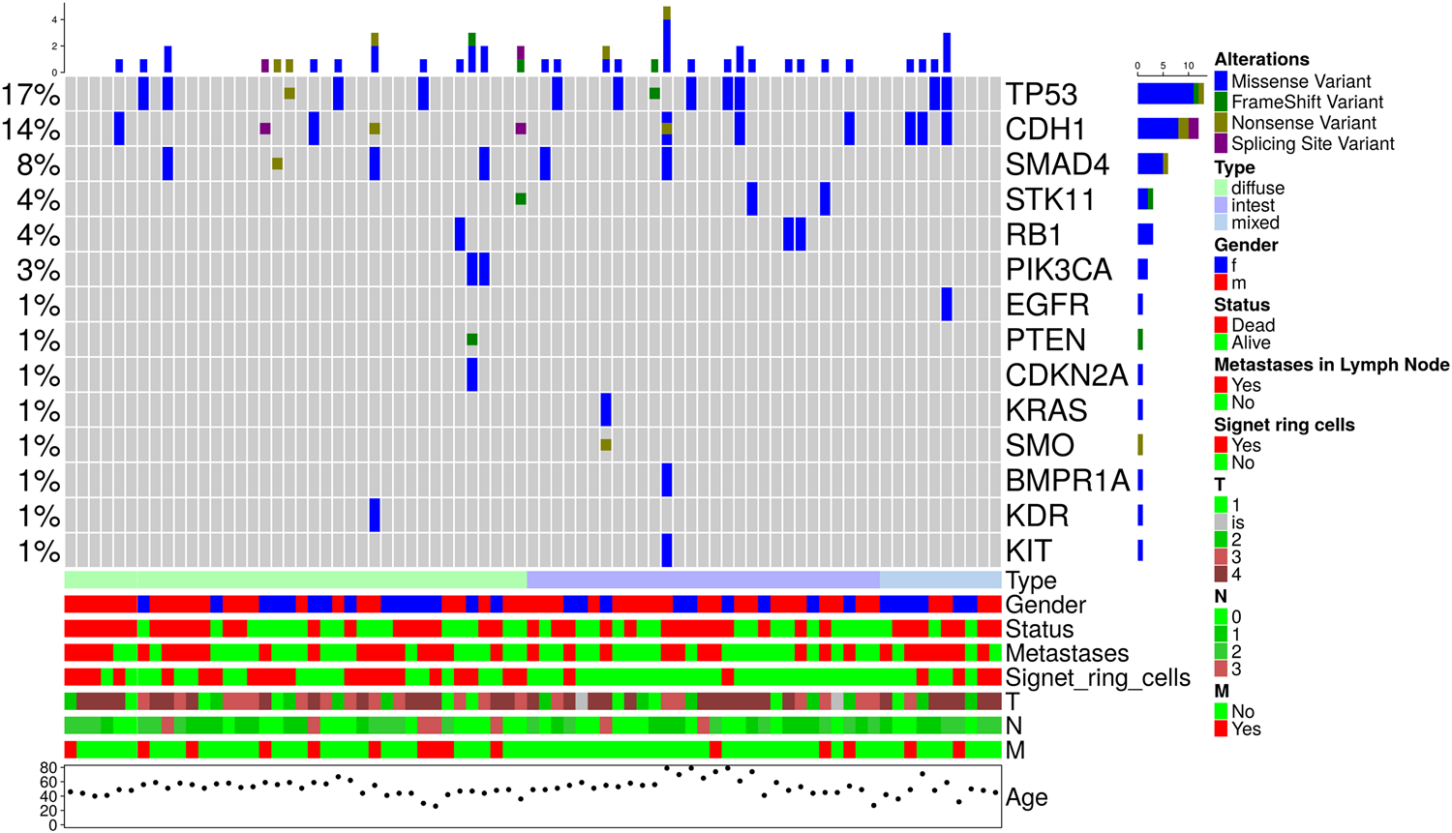
Distribution of mutations in the 77 gastric tumours under study. Each column denotes an individual tumour, and each row represents a gene. Only genes with at least one mutation found in our cohort are depicted.

In the tumour samples of 32/77 (42%) patients, we identified at least one somatic mutation, whereas no mutations that met the selection criteria were found in the remaining 45/77 (58%) patients (Fig. 1). We found no associations of overall tumour somatic mutational status (absence of mutations in the genes under study *vs* presence of at least one mutation) with patients’ age, gender, 5-year overall survival, lymph node metastases and distant metastases or such tumour characteristics as size, stage, Lauren type and presence of signet ring cells (Table 3).

**Table 3:** Clinical significance of overall somatic mutational status and selectively of somatic mutations in the *CDH1* and *TP53* genes in patients with gastric cancer.

	Number of cases	mut+	*Р value*	mut−	CDH1 mut+	*Р value*	TP53 mut+	*Р value*
Total number of patients	77	32		45	11		13	
Men Women	47 30	17 15	0.24	30 15	5 6	0.32	7 6	0.55
Age 27–49 50–79 27–45	35 42 21	13 19 8	0.49	22 23 13	7 4 4	0.21	5 8 3	0.76
T1-2 T3-4 cis	21 54 2	6 25 1	0.36	15 29 1	3 7 1	0.33	2 11 0	0.43
Lymph node metastases N0 N1-3	28 49	11 21	0.81	17 28	2 9	0.31	5 8	1.0
Metastasis No Yes	41 36	14 18	0.17	27 18	2 9	***0.019***	5 8	0.36
Stage I-II III-IV	29 48	8 24	0.06	21 24	2 9	0.19	3 10	0.34
5-year survival status Dead Alive N/A (onset less than 5 years ago)	41 28 7	21 9 2	0.17	20 19 6	6 4 1	0.99	9 3 1	0.44
Loren classification Diffuse Intestinal Not classified	38 29 10	13 15 4	0.35	25 14 6	5 3 3	0.29	5 7 1	0.4
Signet ring cells Yes No	27 50	10 22	0.63	17 28	7 4	***0.043***	3 10	0.52

We further investigated the clinical significance of somatic mutations in the *CDH1* and *TP53* genes in patients with GC. The results are presented in Table 3. We found no significant differences in the groups with *CDH1* and *TP53* mutations regarding gender, age, tumour localization, lymph node metastasis, distant metastasis, stage, and Lauren type. As expected, somatic *CDH1* mutations were positively correlated with distant metastases (p = 0.019). *CDH1* mutations were also observed significantly more frequently in tumours with signet ring cells (p = 0.043).

To investigate the prognostic value of the detected mutations, we conducted a study of the overall survival (OS) of GC patients within the 5-year interval after surgery. OS associations with tumour mutational status were studied in groups with or without lymph node metastasis, distant metastasis, different tumour stages (I-II *vs* III-IV), Lauren classification as diffuse or intestinal, gender and age.

**Figure 2:**
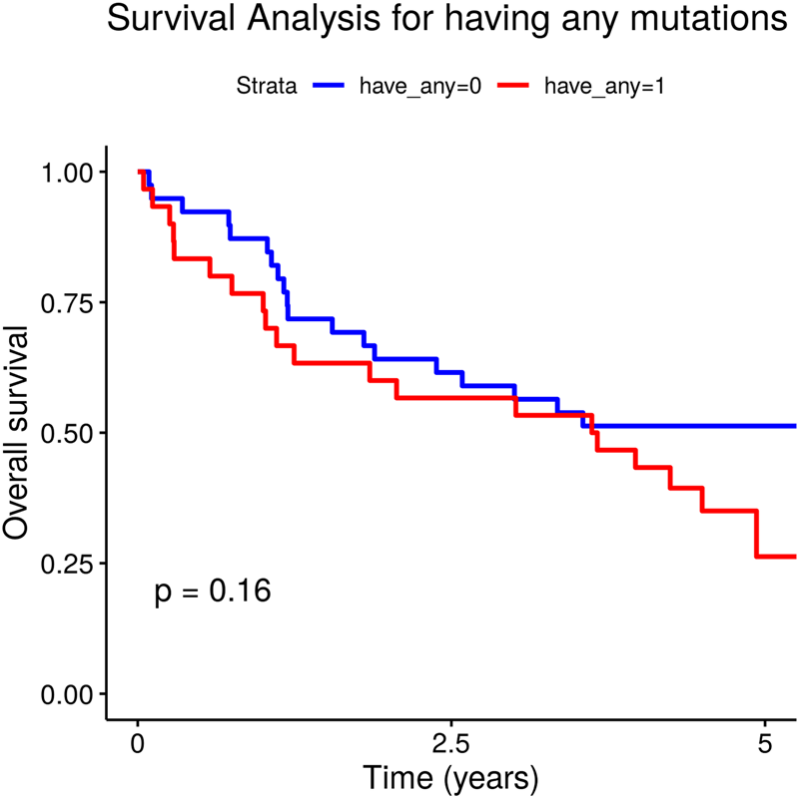
Absence of association between tumour mutational status and overall survival in GC patients. The blue graph describes a subcohort of GC patients with at least one somatic mutation, and the red graph is for the patients with no somatic mutations identified in the genes under study.

Regarding the overall tumour somatic mutational status, we detected no difference in OS of patients carrying at least one mutation in the genes under study *vs* those with no mutations in the same genes (Fig. 2). OS assessed on the whole CG patient cohort under study was also independent of somatic mutation to either the *CDH1* gene or the *TP53* gene, which is in line with TCGA Provisional data estimated by cBioPortal.

**Figure 3:**
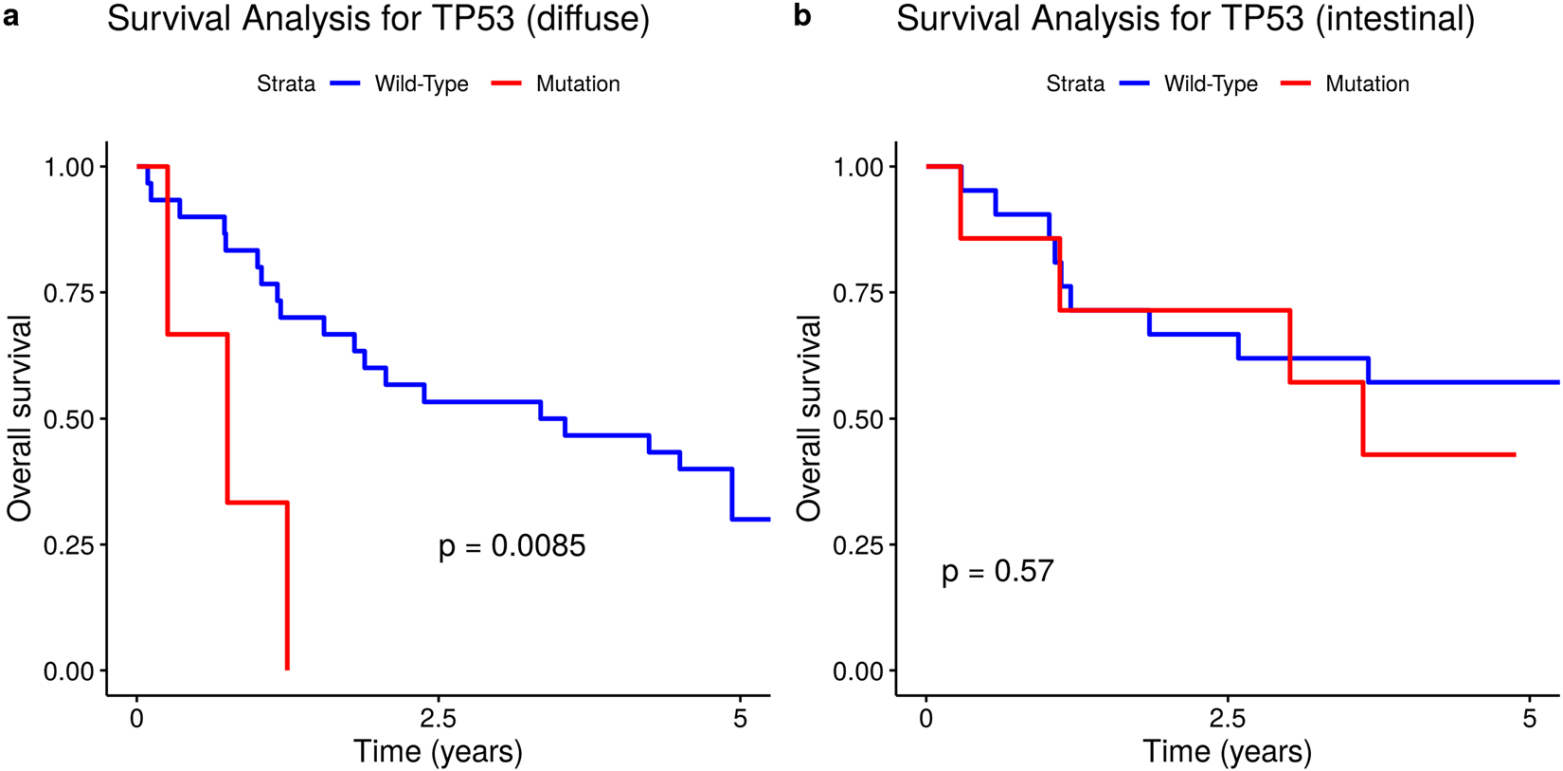
Somatic *TP53* mutational status and overall survival in GC patients with diffuse and intestinal Lauren tumour types. (**a)** Diffuse type. (**b)** Intestinal type.

**Figure 4:**
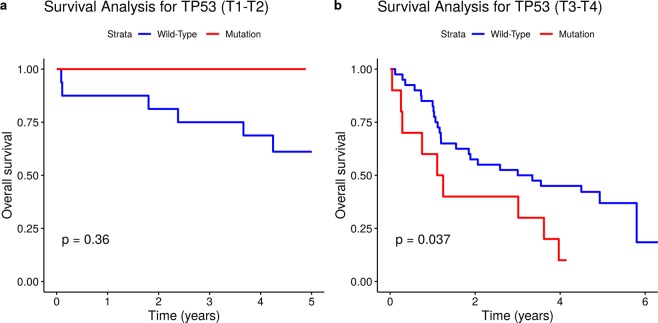
Somatic *TP53* mutational status and overall survival in GC patients with T1-T2 tumours (**a**) and with T3-T4 tumours (**b**).

**Figure 5:**
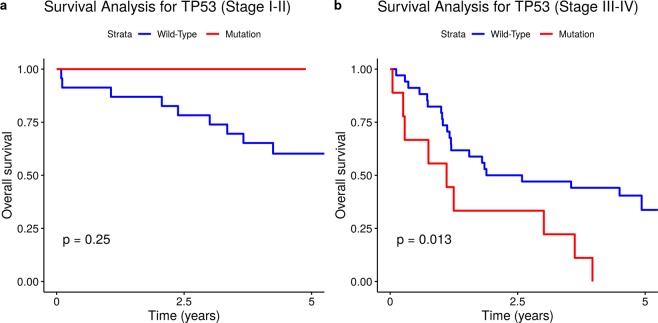
Somatic *TP53* mutational status and overall survival in GC patients with different tumour stages. **(a)** Stages I-II. **(b)** Stages III-IV.

We further analysed OS in different clinical groups with respect to the presence of *TP53* and *CDH1* mutations in tumours. OS appeared to be significantly decreased in the groups of patients with tumours harbouring *TP53* mutations with diffuse Lauren type (p < 0.0085; Fig. 3a), with T3-T4 tumours (p = 0.037; Fig. 4b), and with stage III-IV tumours (p = 0.013; Fig. 5b).

### Evaluation of pathogenicity for genetic variants not annotated in human mutation databases

The pathogenicity of 25 missense genetic variants that were either identified in our study for the first time, had been previously reported in populations but lacked annotations in the human mutation databases, or were ambiguously annotated in terms of clinical significance (conflicting interpretations were presented regarding pathogenicity or uncertain significance), was assessed using the prediction programs PolyPhen2, PROVEAN, SNPs&GO and MutPred2, I-Mutant 3.0 and MutPred-LOF. The results are presented in Supplementary Tables 1 and 2.

According to the PolyPhen2 HumDiv, 6 substitutions are benign; among these substitutions, only one is in *CDH1*, and one is in *TP53*, while others may be damaging. PolyPhen2-HumVar classified 10 substitutions as benign, but it should be borne in mind that this program is better in predicting pathogenicity for Mendelian disorders. PROVEAN indicates that 16 substitutions are deleterious, and 9 of them are neutral. SNPs&GO indicates that 22/25 substitutions are pathogenic with a different reliability index.

MutPred2 and MutPred-LOF software predict how single-nucleotide substitutions can affect the molecular mechanisms in a cell. In our set of somatic genetic variants obtained from gastric tumours, MutPred2 and MutPred-LOF predicted significant disruption of various molecular mechanisms for 13/25 variants with p-values < 0.05 (Supplementary Table 2). The I-Mutant 3.0 tool predicts a decrease in protein stability for 23/25 mutant variants, whereas for two mutations (CDH1:c.A1199T:p.4. D400V and TP53:c.517 G > T:p. V173L), the stability of a mutant protein molecule was predicted to be increased.

The results of this study indicate that the assessed substitutions may be considered to be pathogenic based on the estimates provided by the bioinformatic tools generally used to predict the effects of genetic variants.

## Discussion

In our cohort of gastric tumours, somatic mutations were most frequently observed in the *CDH1* and *TP53* genes. Mutations in these genes were previously described as the most frequent in other studies, although the reported percentages vary between cohorts^7,8^. Discrepancies in the reported frequencies of the mutations detected in these genes may be explained by different approaches to attribution of a genetic variant to the list of deleterious mutations, as well as by ethnic characteristics of the patients. It was demonstrated that the frequencies of somatic mutations of certain genes (e.g., *APC, ARID1A, KMT2A, PIK3CA* and *PTEN*) differ between Caucasian and Asian GC patients^9^. According to TCGA data, *CDH1* mutations were identified in 11% of all GCs, and *ТР53* mutations were determined in 50% of non-hypermutated GCs and in 71% of chromosomally unstable (CIN) samples^10^.

In our study, we used two NGS panels to screen for somatic mutations in GS samples. One panel is the Ion AmpliSeq Cancer Hotspot Panel v2, which has been previously used for GC genotyping in a clinical cancer research^11^. Using this panel, we identified 11 mutations in 8 of the 50 genes studied in our 77 GC samples. Additionally, 36 mutations were identified using a Hereditary Gastric Cancer Panel (HGC), designed by our group, with full coverage of the coding regions of the *TP53, CDH1, PTEN, BMPR1A, SMAD4* and *STK11* genes associated with the development of hereditary GC. All identified mutations were located in the driver genes for GC. The application of our oligogene NGS panel for mutational profiling of GC may enable identification of hereditary cases in clinical practice. Furthermore, detection of *CDH1* and *TP53* mutations can serve as a surrogate marker to distinguish the chromosomally unstable GC, which is enriched for *TP53* mutations, from the genomically stable subtype, which is enriched for *CDH1* mutations^3^. In addition, this test may assist in distinguishing MSS-TP53+ and MSS-TP53−, as well as MSS-ETM, enriched for *CDH1* mutations, when used for ACRG (Asian Cancer Research Group) GC type detection^4^.

The pivotal role played by *CDH1* mutations in the development of GC is undoubted^12^. In our study, somatic *CDH1* mutations were positively correlated with distant metastases (p = 0.019). *CDH1* mutations were also significantly more frequent in tumours with signet ring cells (p = 0.043). However, we have identified no independent prognostic value of somatic *CDH1* mutations, which is in line with TCGA Provisional data estimated by cBioPortal.

Inactivation of *CDH1* during GC development occurs in the tumour because of genetic and epigenetic changes. Loss of *CDH1* expression appears after inactivation of both copies, where one allele can be mutant, and the second allele is inactivated by promotor methylation in approximately 50% of cases. We have previously demonstrated that *CDH1* promoter methylation is associated with the diffuse Lauren type and locally advanced GC^13^. Other studies have shown the prognostic relevance of *CDH1* mutations in diffuse GC, where the presence of somatic mutations in this gene was associated with a decrease in patient survival, regardless of the stage of the disease^14^.

In our cohort, a group of 35 patients had early manifestation of GC, age of disease onset ranging from 26 to 49 years, and no family cancer history. We assumed that this group of patients might harbour a certain mutation spectrum or enrichment with germline mutations in the genes associated with the development of hereditary GC. However, we found no significant differences in the somatic mutation profile and no enrichment with germline mutations for this group. Cho *et al*. found a significant increase in the frequency of somatic mutations of *CDH1* and *TGFBR1* in patients from Korea with early manifestations of GC before 45 years (P < 0.001 for *CDH1* and P = 0.014 for *TGFBR1*)^15^. We assessed data for patients with manifestations of GC before 45 years in our cohort and found no significant increase in the frequency of *CDH1* somatic mutations in this group.

Currently, patients with early manifestations of GC (before 45–50 years) are classified into an independent cancer group of early-onset GC (EOGC) with specific clinical and molecular characteristics. This group features prevalence among women, multifocal growth and diffuse phenotype of the tumour without intestinal metaplasia. A molecular profile of the tumour is characterized by the absence or low level of microsatellite instability (MSI), rare loss of heterozygosity (LOH), retained expression of *RUNX3*, amplifications of 17q, 19q and 20q, and high expression of low-molecular-weight cyclin E isoforms^16^. The reported prognosis for this group varies from better to poorer survival depending on the study^17,18^. Our study did not reveal a statistically significant difference in OS for patients with early GC onset, but we detected a decrease in the survival rate of patients in this group in the presence of somatic *TP53* mutations.

Although *TP53* mutations in GC have long been studied, the clinical relevance of these mutations for the prognosis of the disease and treatment of patients has not been fully determined. A large number of studies have presented diametrically opposite results, which may be explained by the specific characteristics of patient cohorts, including the ethnicity of the studied groups. At present, the frequency of mutant alleles of this gene is used to stratify patients into molecular subtypes, to predict the course of the disease and to control the response to chemotherapy. It was demonstrated that the mutant allele frequency (AF) decreases during chemotherapy in GC patients^19^.

Discrepancies in the estimations of the clinical significance of the somatic mutations presented by different research teams may not only be caused by ethnic factors but also by differences in the criteria used to attribute a genetic variant to a class of pathogenic or possibly pathogenic mutations. Standards and guidelines for the interpretation of sequence variants widely used in medical genetics, such as those provided by the American College of Medical Genetics and Genomics and the Association for Molecular Pathology^20^, cannot be directly extrapolated to cancer somatic variants. *In silico* predictors of pathogenicity for missense variants are 65–80% accurate when examining known disease variants, and some are intended for analysis of Mendelian disorders^20^. In our study, we adopted the extremely low general population frequency of a genetic variant as a key criterion of its inclusion in the downstream analysis of the clinical significance of somatic mutations in a cohort of GC patients. More specifically, genetic variants with MAF > 0.0001 were excluded from analysis, which means that the remaining alternative alleles were either never found in gnomAD or were observed extremely rarely. At present, this criterion may be considered rather stable; with at least 60,000 human exomes annotated^21^, we are not expecting dramatic fluctuations in allele frequencies in the human population in the foreseeable future.

## Materials and Methods

### Patients and tumour samples

The study included 77 patients with locally advanced GC who were treated in N.N. Burdenko Facultative Surgery Clinic, I.M. Sechenov First Moscow State Medical University from 2007 to 2015. This study was conducted in accordance with the Declaration of Helsinki and was approved by the Institutional Ethics Committee of I.M. Sechenov First Moscow State Medical University. Written informed consent was obtained from each participant in this study. All patients underwent surgical treatment, and resected tumour samples were used in the study. GC was confirmed in all patients by morphological examination of the surgical material. According to the Lauren classification, intestinal GC was confirmed in 29/77 cases, and diffuse GC was confirmed in 38/77 cases. The distribution of patients in clinical groups is presented in Table 4.

**Table 4:** Clinicopathological parameters of gastric cancer patients, n = 77.

	Number of cases (%)
Total number of patients	77 (100)
Men Women	47 (61,1) 30 (38,9)
Age 27–49 50–79	35 (44,2) 43 (55,8)
T1-2 T3-4 cis	21(27,5) 54 (70) 2 (2,5)
Lymph node metastases N0 N1-3	28 (36,4) 49 (63,6)
Distant metastases No Yes	41(53,2) 36 (46,7)
Stage I II III IV	11 (14,2) 18 (23.3) 33 (42,8) 15 (19,4)
5-year survival status Dead Alive N/A (onset less than 5 years ago)	41 (53,2) 28 (36,4) 7 (9,1)
Lauren classification Diffuse Intestinal Mixed	38 (49,3) 29 (37,6) 10 (12,9)
Signet ring cells Yes No	27 (35,0) 50 (64,9)

### Mutation screening by NGS

Five to seven 10-μm paraffin sections were manually dissected to ensure that each tumour sample contained at least 70% of neoplastic cells. Genomic DNA was isolated from archived samples using a QIAamp DNA FFPE Tissue kit, as recommended by QIAGEN (Germany).

Deep sequencing was performed using the Ion Torrent platform (Life Technologies) following established protocol^22^. The protocol includes the preparation of libraries of genomic DNA fragments, clonal emulsion PCR, sequencing and bioinformatic analysis of results. DNA fragment libraries were prepared using Ion Ampliseq ultra-multiplex PCR technology.

A commercially available oligonucleotide panel Ion AmpliSeq Cancer Hotspot Panel v2 (Life Technologies) was used as a set of multiplex primers. The panel consists of 207 primer pairs for simultaneous amplification of mutation hotspots in 50 cancer-related genes (in alphabetical order): *ABL1, AKT1, ALK, APC, ATM, BRAF, CDH1, CDKN2A, CSF1R, CTNNB1, EGFR, ERBB2, ERBB4, EZH2, FBXW7, FGFR1, FGFR2, FGFR3, FLT3, GNA11, GNAS, GNAQ, HNF1A, HRAS, JAK2, JAK3, IDH1, IDH2, KDR/VEGFR2, KIT, KRAS, MET, MLH1, MPL, NOTCH1, NPM1, NRAS, PDGFRA, PIK3CA, PTEN, PTPN11, RB1, RET, SMAD4, SMARCB1, SMO, SRC, STK11, TP53*, and *VHL*^23^.

Additionally, we utilized our original, customized panel, comprised of six hereditary GC-related genes (HGC panel). An HGC panel with 218 primer pairs was designed to amplify all coding regions, noncoding regions of the terminal exons, and putative splice site gene regions for six human genes: *BMPR1A, SMAD4, CDH1, TP53, STK11*, and *PTEN*. The panel was designed using the Ion Ampliseq Designer v.3.6, which minimizes the number of oligonucleotide pair pools that are necessary to completely cover the target genomic sequences. The total length of human genome sequences covered by the HGC panel is 42,320 bp.

Multiplex PCR and subsequent stages of the fragment library preparation were undertaken using an Ion AmpliSeq Library Kit 2.0 (Life Technologies) according to the manufacturer’s protocol. Aliquots from the prepared libraries were subjected to clonal amplification on microspheres in the emulsion on the Ion OneTouch instruments using the Ion OneTouch 200 Template Kit v2 DL (Life Technologies). Effective products of the emulsion PCR, the microspheres coated with the target amplicons, were purified from empty microspheres on the Ion OneTouch Enrichment System. Sequencing was performed on the Ion Torrent PGM genomic sequencer using an Ion PGM 200 Sequencing Kit (Life Technologies). The results were analysed with Torrent Suite software consisting of Base Caller (the primary analysis of the sequencing results); Torrent Mapping Alignment Program - TMAP (alignment of the sequences to the reference genome GRCh37/hg19); and Torrent Variant Caller (analysis of variations in nucleotide sequences). Genetic variants were annotated with ANNOVAR software^24^. Visual data analysis, manual filtering of sequencing artefacts and sequence alignment were performed using the Integrative Genomics Viewer (IGV)^25^.

To investigate the hereditary cancer syndrome genes involved in Li-Fraumeni syndrome, Peutz–Jeghers syndrome, Cowden syndrome, hereditary GC and hereditary gastrointestinal polyposis, we designed a panel consisting of 218 primer pairs for PCR amplification of all coding sequences of the human genes *BMPR1A, SMAD4, CDH1, TP53, STK11* and *PTEN*, as well as exon flanks and terminal untranslated regions (UTRs). A primer panel was designed with Ion Ampliseq Designer v.3.6 software. The total length of human genome sequences covered by this panel is 42320 bp.

### Validation of mutations detected by next-generation sequencing

Validation of the mutations detected by NGS screening was performed to exclude sequencing artefacts in tumour samples. The direct sequencing of individual PCR products from primers that flank areas of specific mutations was performed on the automatic genetic analyser ABI PRISM 3500 (Life Technologies, Carlsbad, United States) according to the manufacturer’s protocols.

### Statistical analysis

Samples were compared using Fisher’s exact test. OS was calculated by the Kaplan–Meier product limit method from the date of surgery until death by any cause. Statistica 7.0 software was used for data processing. The results obtained from our tumour tissue collection were compared to the most comprehensively characterized collection of stomach adenocarcinoma samples (TCGA, Provisional) with the assistance of The cBioPortal (www.cbioportal.org), an open access resource for interactive exploration of multidimensional cancer genomics datasets^26^.

### Pathogenicity prediction for novel mutations

To predict the pathogenicity of identified novel missense variants, PolyPhen2, PROVEAN, SNPs&GO and MutPred2 tools were used. I-Mutant 3.0 software was used to calculate the stability of the mutant protein. Loss of protein function effects were assessed with MutPred-LOF software. The effect of nonsynonymous substitutions on the structure was illustrated using the Project HOPE3D portal.

## Supplementary information

Supplementary Information.

## Data Availability

Raw sequence reads data are available at the NCBI BioProject, Accession: PRJNA560649, ID: 560649 (https://www.ncbi.nlm.nih.gov/bioproject/560649).
