# Supplementary material for: Clinical relevance of somatic mutations in main driver genes detected in gastric cancer patients by next-generation DNA sequencing
Source: Sci Rep. 2020 Jan 16;10:504. doi: 10.1038/s41598-020-57544-3 (PMC6965114; doi:10.1038/s41598-020-57544-3)
Supplement: Supplementary file 1 — Supplementary Information. [file 41598_2020_57544_MOESM1_ESM.docx]

**Clinical relevance of somatic mutations in main driver genes detected in gastric cancer patients by next-generation DNA sequencing**

**Marina V Nemtsova^1,2^, Alexey I Kalinkin^2^, Ekaterina B Kuznetsova^1,2^, Irina V Bure^1^, Ekaterina A Alekseeva^1,2^, Igor I Bykov^3^, Tatiana V Khorobrykh^3^, Dmitry S Mikhaylenko^1,2,4^, Alexander S Tanas^2^, Sergey I Kutsev^2^, Dmitry V Zaletaev^1,2^ & Vladimir V Strelnikov^2,*^**

^1^Medical Genetics Laboratory, I.M. Sechenov First Moscow State Medical University, Moscow, 119991, Russian Federation.

^2^Epigenetics Laboratory, Research Centre for Medical Genetics, Moscow, 115522, Russian Federation.

^3^Department No 1, Medical Faculty, Faculty Surgery, I.M. Sechenov First Moscow State Medical University, Moscow, 119991, Russian Federation.

^4^N.A. Lopatkin Research Institute of Urology and Interventional Radiology – branch of the National Medical Research Radiologiсal Center, Moscow, 105425, Russian Federation.

*Author for correspondence: [vstrel@list.ru](mailto:vstrel@list.ru)

**Sup Fig. S1**

**
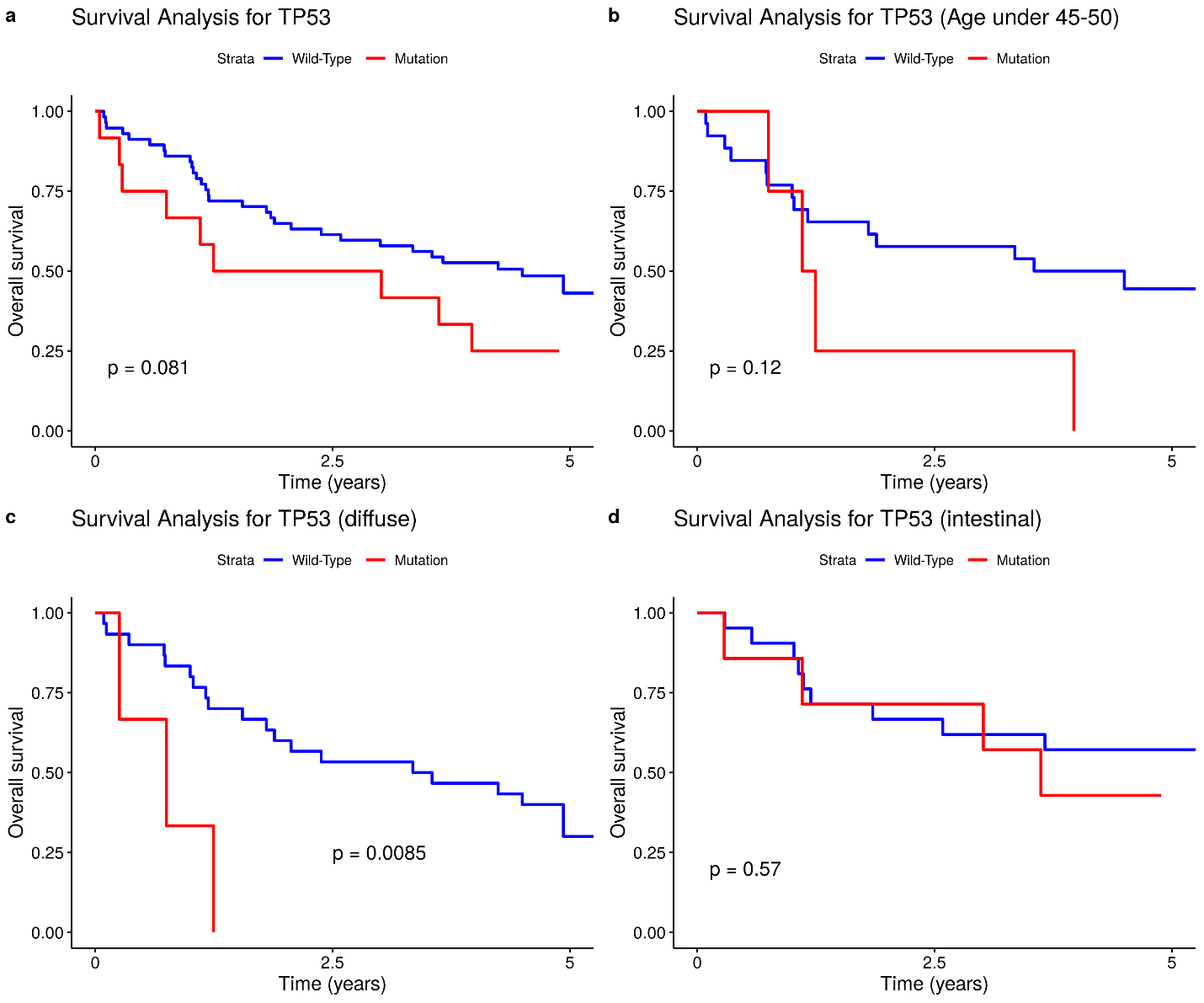
**

**Sup Figure S1.** Kaplan-Meier overall survival curves for the gastric cancer patients with and without somatic *TP53* mutations, with respect to age of onset and Lauren tumor types. (**a)** All the patients under study. **(b)** Patients under 45. **(c)** Diffuse Lauren type. **(d)** Intestinal Lauren type.

**Sup Fig. S2**

| **a**  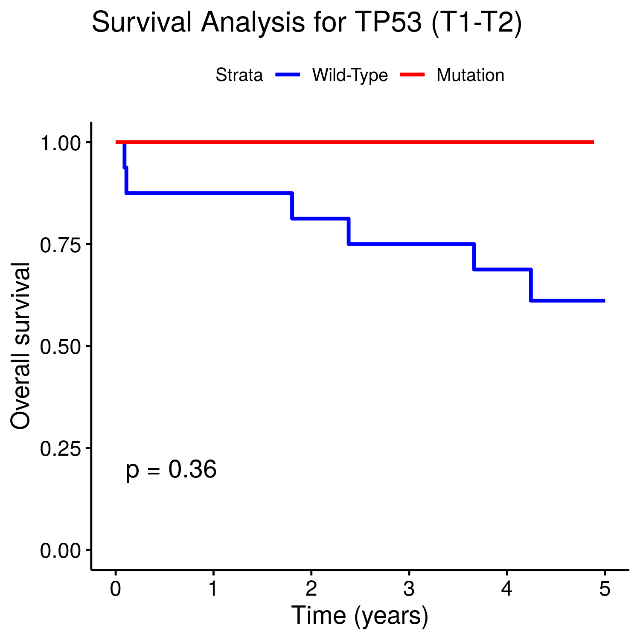 | **b**  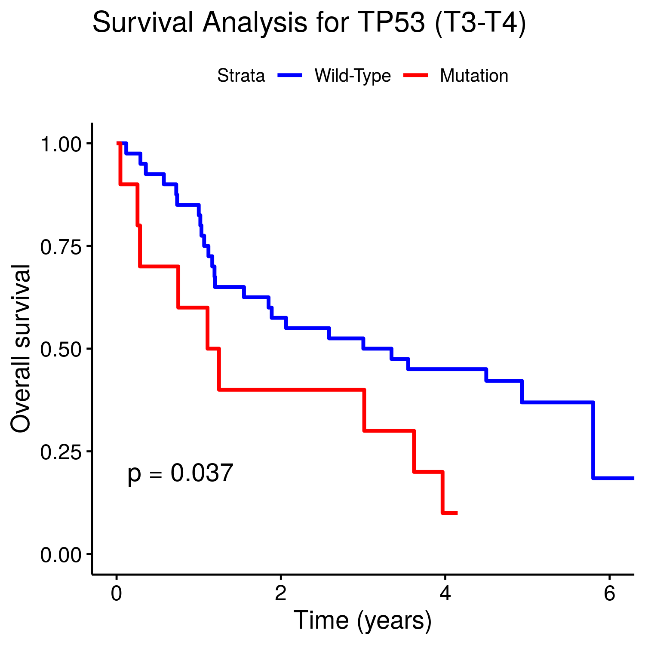 |
| --- | --- |
| **c**  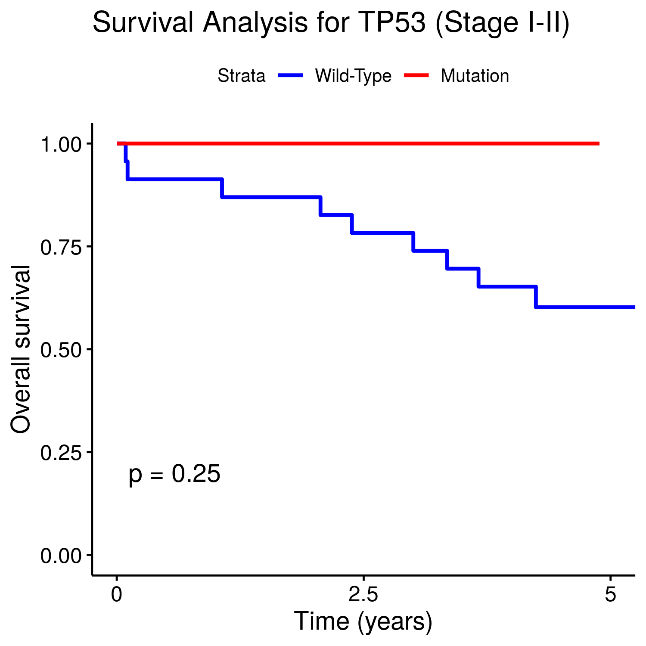 | **d**  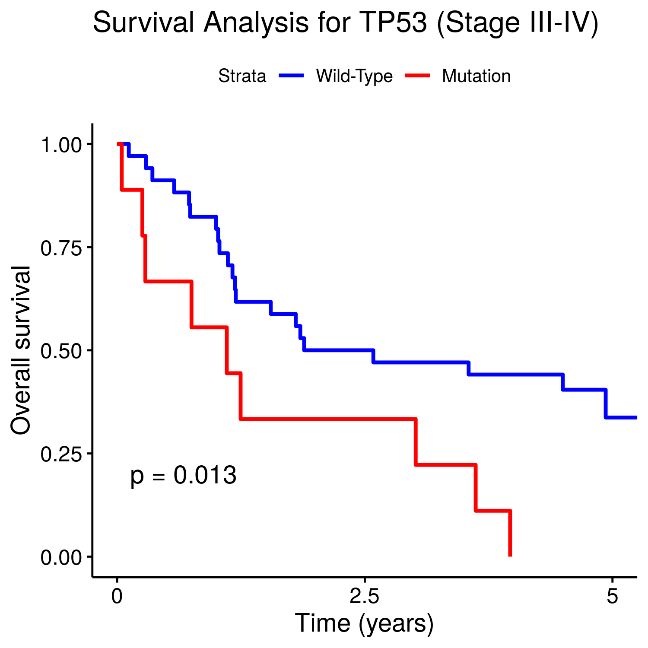 |

**Sup Figure S2.** Kaplan-Meier overall survival curves for the gastric cancer patients with and without somatic *TP53* mutations, with respect tumor size and stages.

**Sup Fig. S3**


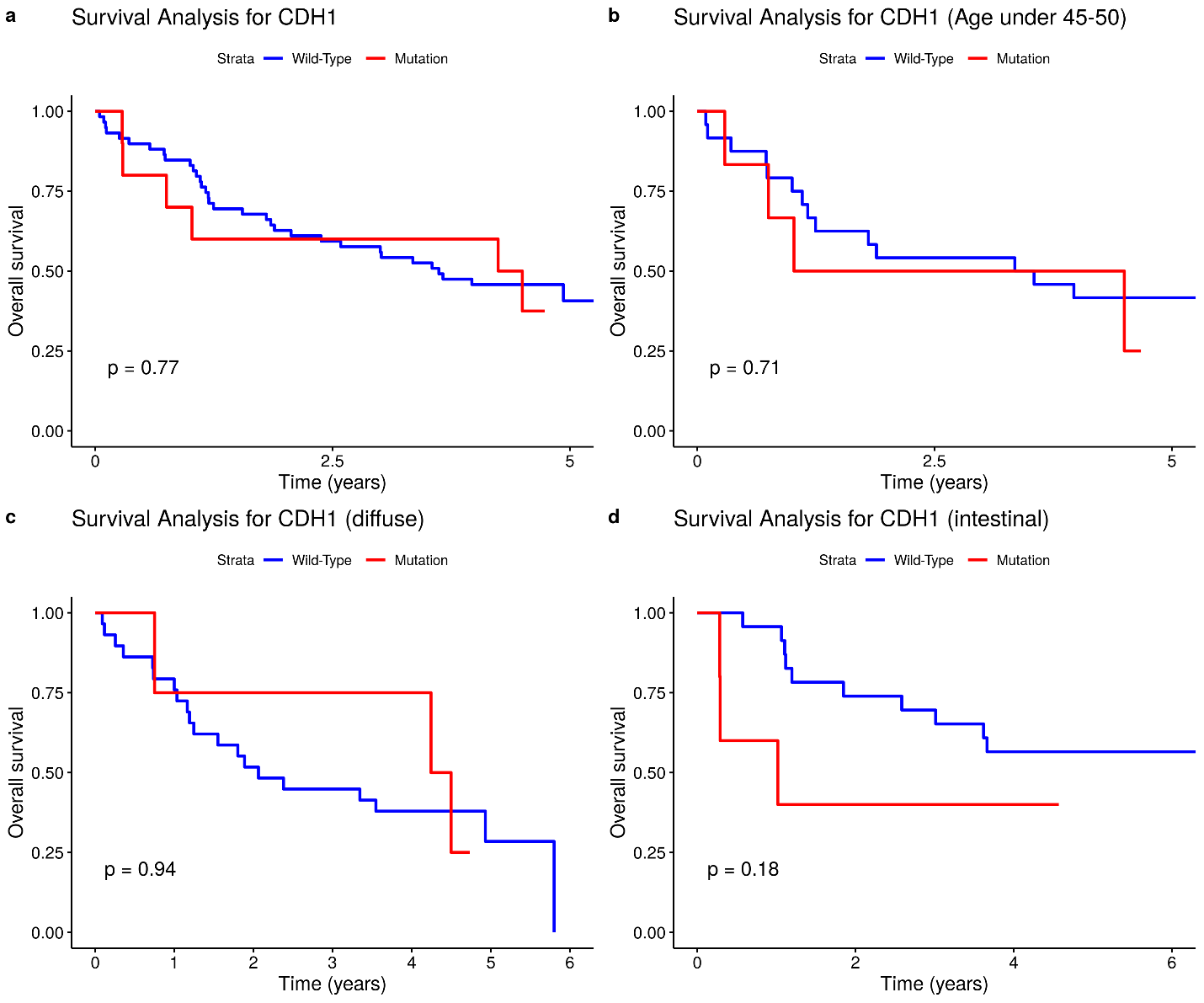


**Sup Figure S3.** Kaplan-Meier overall survival curves for the gastric cancer patients with and without somatic *CDH1* mutations, with respect to age of onset and Lauren tumor types. (**a)** All the patients under study. **(b)** Patients under 45. **(c)** Diffuse Lauren type. **(d)** Intestinal Lauren type.

**Sup Fig. S4**

| **a**  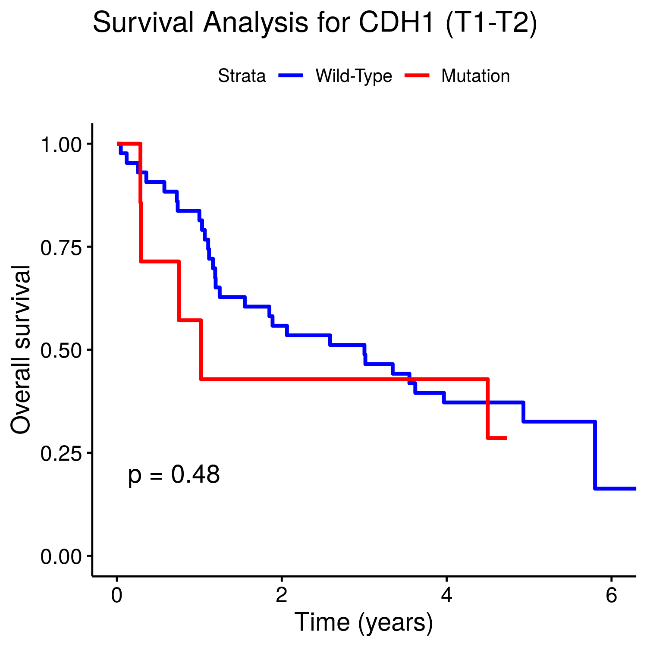 | **b**  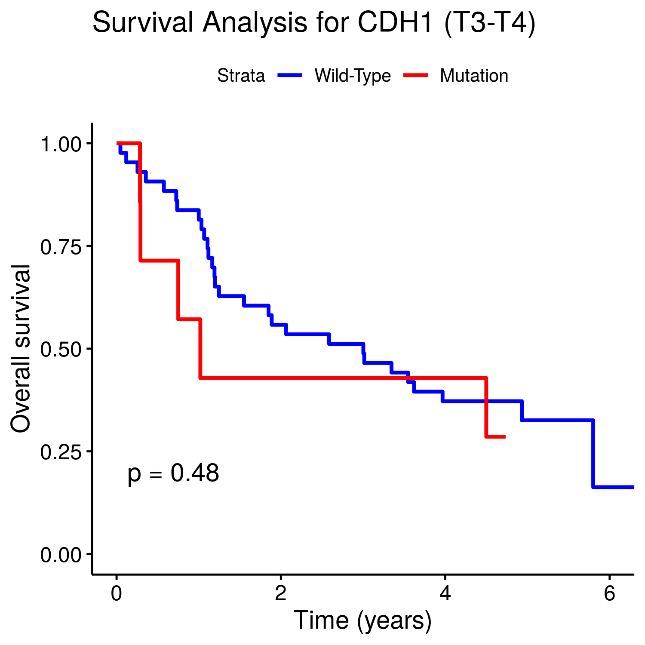 |
| --- | --- |
| **c**  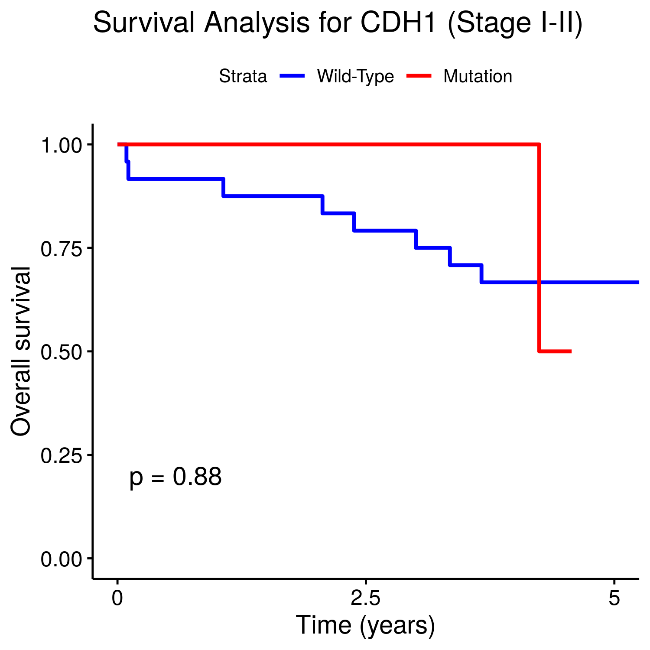 | **d**  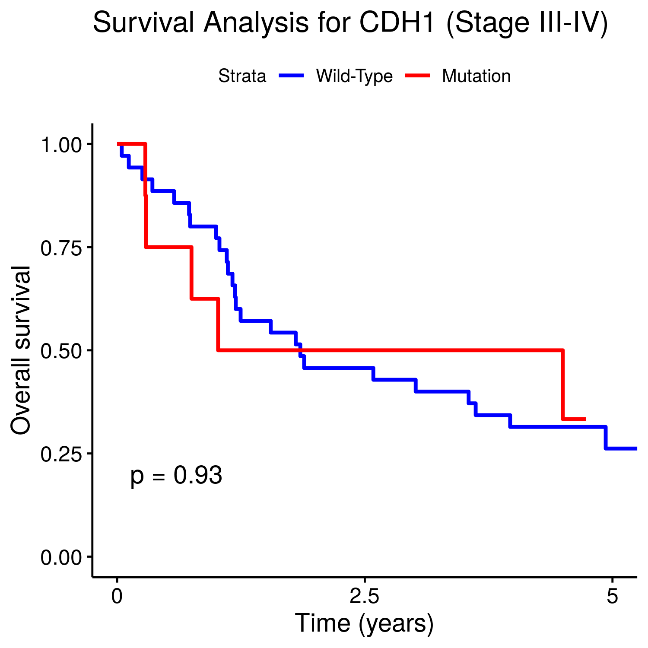 |

**Sup Figure S4.** Kaplan-Meier overall survival curves for the gastric cancer patients with and without somatic *CDH1* mutations, with respect tumor size and stages.

**Supplementary Table S1.** Evaluation of pathogenicity for missense somatic genetic variants identified in this study

|  | General Information | | | PolyPhen2 | | | | PROVEAN | | SNPs&GO | | I-Mutant 3.0 | | MutPred2 |
| --- | --- | --- | --- | --- | --- | --- | --- | --- | --- | --- | --- | --- | --- | --- |
|  | Gene | Genetic Variant | Type | Hum  Div Prob. | Hum  Div  Pred. | Hum  Var Prob. | Hum  Var  Pred. | Score | Prediction | Reliability Index | Prediction | ddG | Stability | Score |
| 1 | *CDH1* | NM_004360.4:  c.1199A>T:p.D400V | Missense | 1 | Probably Damaging | 1 | Probably Damaging | -8.35 | Deleterious | 9 | Disease | 0.48 | Increase | 0.904 |
| 2 | *CDH1* | NM_004360.4:  c.779C>G:p.P260R | Missense | 1 | Probably Damaging | 1 | Probably Damaging | -8.32 | Deleterious | 8 | Disease | -0.79 | Decrease | 0.799 |
| 3 | *CDH1* | NM_004360.4:  c.1198G>A:p.D400N | Missense | 1 | Probably Damaging | 1 | Probably Damaging | -4.64 | Deleterious | 9 | Disease | -0.18 | Decrease | 0.774 |
| 4 | *CDH1* | NM_004360.4:  c.641T>C:p.L214P | Missense | 1 | Probably Damaging | 1 | Probably Damaging | -5.68 | Deleterious | 9 | Disease | -1.61 | Decrease | 0.943 |
| 5 | *KDR* | NM_002253:  c.G2678C:p.G893A | Missense | 1 | Probably Damaging | 1 | Probably Damaging | -5.29 | Deleterious | 4 | Disease | -0.71 | Decrease | 0.827 |
| 6 | *SMAD4* | NM_005359:  c.1066C>T:p.P356S | Missense | 1 | Probably Damaging | 1 | Probably Damaging | -7.82 | Deleterious | 10 | Disease | -1.65 | Decrease | 0.865 |
| 7 | *TP53* | NM_000546.5:  c. 734G>A:p.G245D | Missense | 1 | Probably Damaging | 0.999 | Probably Damaging | -6.86 | Deleterious | 10 | Disease | -0.92 | Decrease | 0.705 |
| 8 | *CDH1* | NM_004360.4:  c.641T>A:p.L214Q | Missense | 1 | Probably Damaging | 0.999 | Probably Damaging | -4.54 | Deleterious | 7 | Disease | -1.95 | Decrease | 0.895 |
| 9 | *STK11* | NM_000455.4:  c.866T>A:p.M289K | Missense | 1 | Probably Damaging | 0.999 | Probably Damaging | -5.30 | Deleterious | 2 | Disease | -1.12 | Decrease | 0.881 |
| 10 | *EGFR* | NM_005228.3:  c.874G>A:p.V292M | Missense | 1 | Probably Damaging | 0.995 | Probably Damaging | -2.59 | Deleterious | 10 | Disease | -1.12 | Decrease | 0.713 |
| 11 | *RB1* | NM_000321.2:  c.2056C>A:p.H686N | Missense | 1 | Probably Damaging | 0.991 | Probably Damaging | -4.42 | Deleterious | 10 | Disease | -0.2 | Decrease | 0.508 |
| 12 | *CDKN2A* | NM_000077.4:  c.307C>T:p.R103W | Missense | 1 | Probably Damaging | 0.964 | Probably Damaging | -5.10 | Deleterious | 10 | Disease | -0.35 | Decrease | 0.306 |
| 13 | *STK11* | NM_000455:  c.928C>T:p.R310W | Missense | 0.999 | Probably Damaging | 0.651 | Possibly Damaging | -3.24 | Deleterious | 2 | Neutral | -0.12 | Decrease | 0.707 |
| 14 | *TP53* | NM_000546.5:  c. 517G>T:p.V173L | Missense | 0.979 | Probably Damaging | 0.916 | Probably Damaging | -2.92 | Deleterious | 10 | Disease | -0.29 | Increase | 0.363 |
| 15 | *SMAD4* | NM_005359:  c.935C>T:p.P312L | Missense | 0.965 | Probably Damaging | 0.373 | Benign | -2.98 | Deleterious | 8 | Disease | -0.25 | Decrease | 0.794 |
| 16 | *RB1* | NM_000321.2:  c.1690C>T:p.L564F | Missense | 0.904 | Possibly Damaging | 0.205 | Benign | -1.00 | Neutral | 8 | Disease | -1.15 | Decrease | 0.112 |
| 17 | *CDH1* | NM_004360.4:  c.418C>T:p.L140F | Missense | 0.865 | Possibly Damaging | 0.695 | Possibly Damaging | -2.08 | Neutral | 5 | Neutral | -1.34 | Decrease | 0.081 |
| 18 | *CDH1* | NM_004360.4:  с.907A>C:pT303P | Missense | 0.628 | Possibly Damaging | 0.221 | Benign | -2.19 | Neutral | 5 | Disease | -0.68 | Decrease | 0.414 |
| 19 | *CDH1* | NM_004360.4:  c.546A>C:p.K182N | Missense | 0.509 | Possibly Damaging | 0.247 | Benign | -2.48 | Neutral | 1 | Neutral | -0.58 | Decrease | 0.147 |
| 20 | *SMAD4* | NM_005359.5:  c.473T>C:p.V158A | Missense | 0.387 | Benign | 0.156 | Benign | -2.37 | Neutral | 5 | Disease | -1.37 | Decrease | 0.562 |
| 21 | *RB1* | NM_000321.2:  c.2002C>T:p.R668C | Missense | 0.151 | Benign | 0.01 | Benign | -3.26 | Deleterious | 9 | Disease | -0.65 | Decrease | 0.341 |
| 22 | *BMPR1A* | NM_004329:  c.250G>A:p.A84T | Missense | 0.04 | Benign | 0.122 | Benign | -0.06 | Neutral | 7 | Disease | -0.48 | Decrease | 0.671 |
| 23 | *CDH1* | NM_004360.4:  c.2512A>G:p.S838G | Missense | 0.022 | Benign | 0.074 | Benign | -1.47 | Neutral | 7 | Neutral | -1.26 | Decrease | 0.160 |
| 24 | *KIT* | NM_000222.2:  c.G148T:p.V50L | MIssense | 0.019 | Benign | 0.044 | Benign | -0.09 | Neutral | 4 | Neutral | -1.49 | Decrease | 0.094 |
| 25 | *TP53* | NM_000546.5:  c.892G>A:p.E298K | Missense | 0.002 | Benign | 0.003 | Benign | -0.22 | Neutral | 10 | Disease | -0.51 | Decrease | 0.043 |

№ 1-14 Probably Damaging PolyPhen2

№ 15-19 Possibly Damaging/ Benign PolyPhen2

№ 20-25 Benign PolyPhen2

**Supplementary Table S2.** Prediction of molecular mechanisms altered by missense mutations (MutPred2)

| Gene | Genetic Variant | Altered Mechanisms (p < 0.05) |
| --- | --- | --- |
| *TP53* | NM_000546.5:  c. 734G>A:p.G245D | Gain of Loop |
| *BMPR1A* | NM_004329:  c.250G>A:p.A84T | Altered Ordered interface; Altered Metal binding; Altered Transmembrane protein; Altered Stability |
| *KDR* | NM_002253:  c.G2678C:p.G893A | Altered Metal binding |
| *SMAD4* | NM_005359.5:  c.473T>C:p.V158A | Loss of SUMOylation at K159; Gain of Ubiquitylation at K159; Altered Stability; Loss of Sulfation at Y162 |
| *SMAD4* | NM_005359:  c.1066C>T:p.P356S | Gain of Proteolytic cleavage at D351; Gain of Sulfation at Y353 |
| *EGFR* | NM_005228.3:  c.874G>A:p.V292M | Altered Metal binding; Loss of Strand; Gain of Disulfide linkage at C295; Altered Transmembrane protein |
| *STK11* | NM_000455.4:  c.866T>A:p.M289K | Altered Disordered interface; Loss of Loop; Loss of Phosphorylation at Y292; Gain of SUMOylation at M289; Gain of Acetylation at M289; Altered DNA binding; Altered Stability |
| *CDH1* | NM_004360.4:  c.1199A>T:p.D400V | Altered Metal binding; Altered Transmembrane protein; Altered Ordered interface; Loss of Catalytic site at D400; Loss of Relative solvent accessibility; Loss of Ubiquitylation at K397; Loss of Methylation at K397 |
| *CDH1* | NM_004360.4:  c.1198G>A:p.D400N | Altered Metal binding; Altered Transmembrane protein; Altered Ordered interface; Gain of Relative solvent accessibility; Loss of Catalytic site at D400; Loss of Ubiquitylation at K397; Loss of Methylation at K397 |
| *CDH1* | NM_004360.4:  c.779C>G:p.P260R | Altered Transmembrane protein; Altered Ordered interface; Loss of Loop; Altered Metal binding; Loss of B-factor; Gain of Relative solvent accessibility; Gain of Allosteric site at P260; Loss of Ubiquitylation at K259; Loss of Catalytic site at N256 |
| *CDH1* | NM_004360.4:  c.641T>C:p.L214P | Altered Ordered interface; Altered Stability; Altered Transmembrane protein; Loss of Relative solvent accessibility; Altered Metal binding |
| *CDH1* | NM_004360.4:  c.641T>A:p.L214Q | Altered Ordered interface; Altered Stability; Altered Transmembrane protein; Loss of Relative solvent accessibility; Altered Metal binding |
| *STK11* | NM_000455:  c.928C>T:p.R310W | Loss of Intrinsic disorder; Altered Disordered interface; Gain of Allosteric site at W308; Gain of Acetylation at K311; Altered DNA binding |

**Sup Fig. S5**


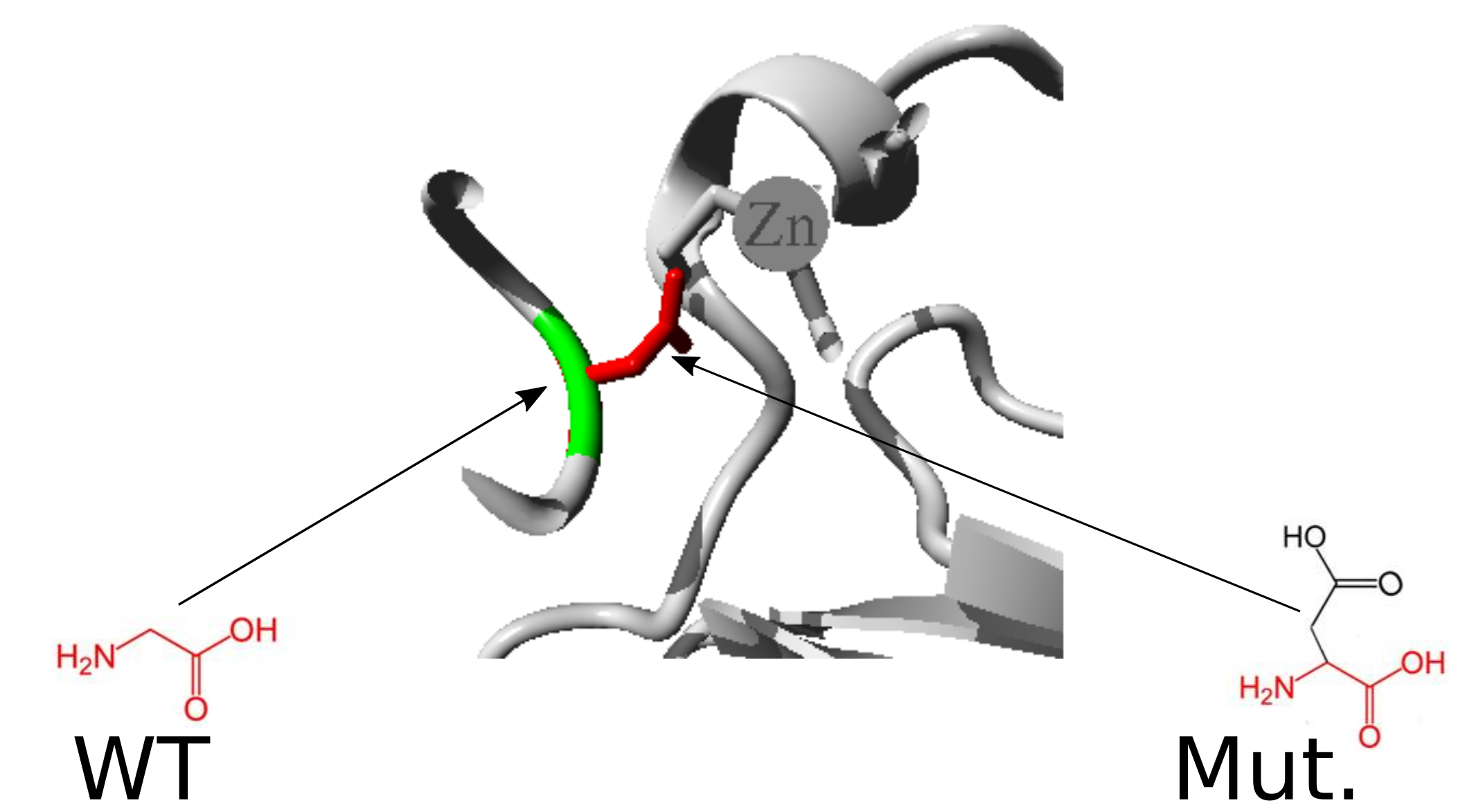


**Sup Fig. S5.** *TP53* (NM_000546.5:c. 734G>A:p.G245D). The mutant residue is bigger than the wild-type residue. The wild-type residue charge was NEUTRAL, the mutant residue charge is NEGATIVE. The wild-type residue is more hydrophobic than the mutant residue. The mutation is located within a stretch of residues annotated in UniProt as a special region: Interaction with CCAR2. The differences in amino acid properties can disturb this region and disturb its function. The wild-type residue is a glycine, the most flexible of all residues. This flexibility might be necessary for the protein's function. Mutation of this glycine can abolish this function. Based on conservation scores this mutation is probably damaging to the protein. The wild-type residue was buried in the core of the protein. The mutant residue is bigger and probably will not fit. The torsion angles for this residue are unusual. only glycine is flexible enough to make these torsion angles, mutation into another residue will force the local backbone into an incorrect conformation and will disturb the local structure.

**Sup Fig. S6**


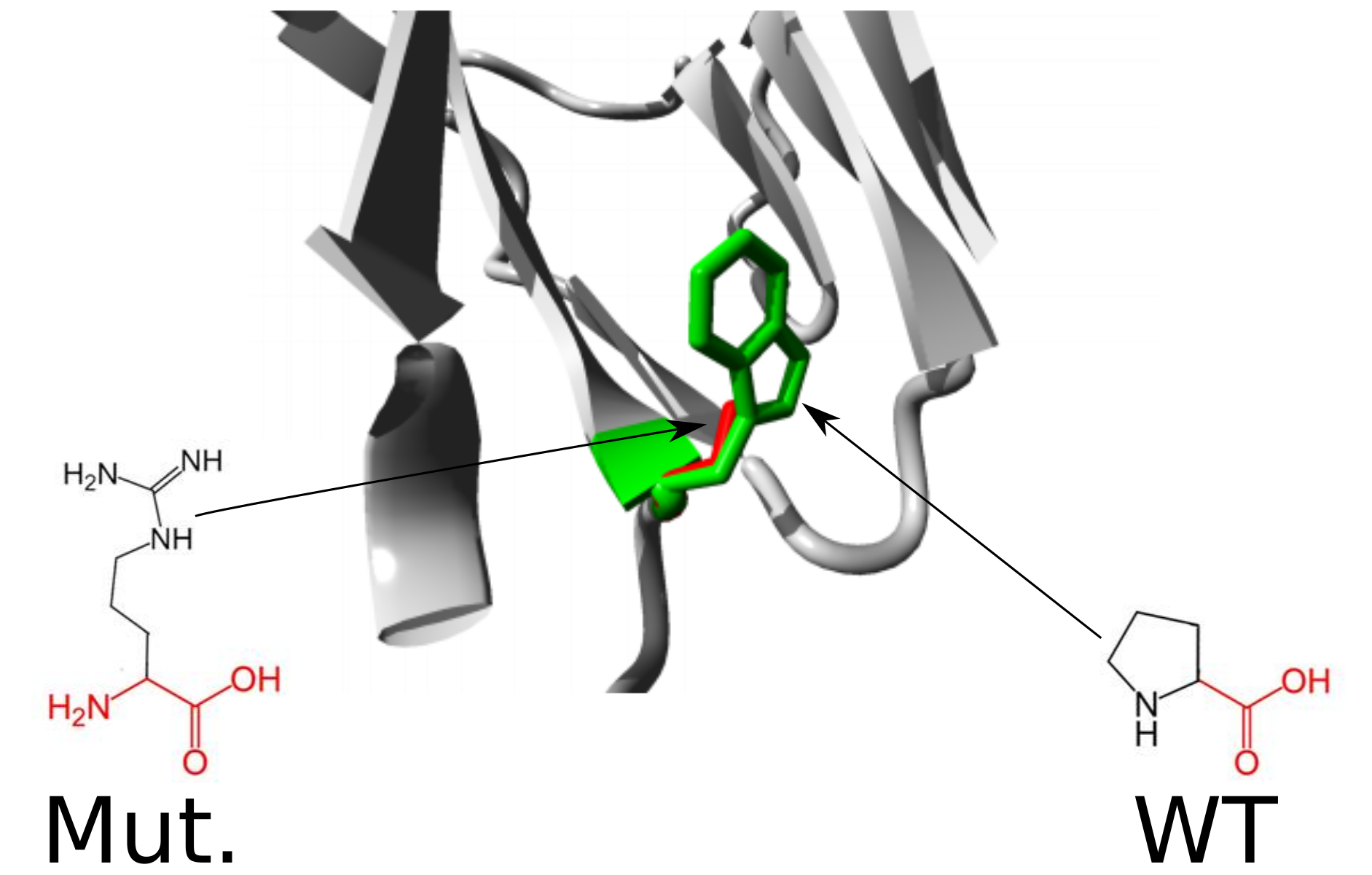


**Sup Fig. S6.** *SMAD4* (NM_005359: c.1066C>T:p.P356S). The modified amino acid is smaller and less hydrophobic than the wild-type amino acid. This replacement is located in the MH2 domain, which may interfere with its functioning. This replacement is located next to the highly conservative area and may be pathogenic. Substitution can disrupt hydrophobic bonds with other molecules on the surface of the protein. This replacement may lead to disruption of external interactions.

**Sup Fig. S7**


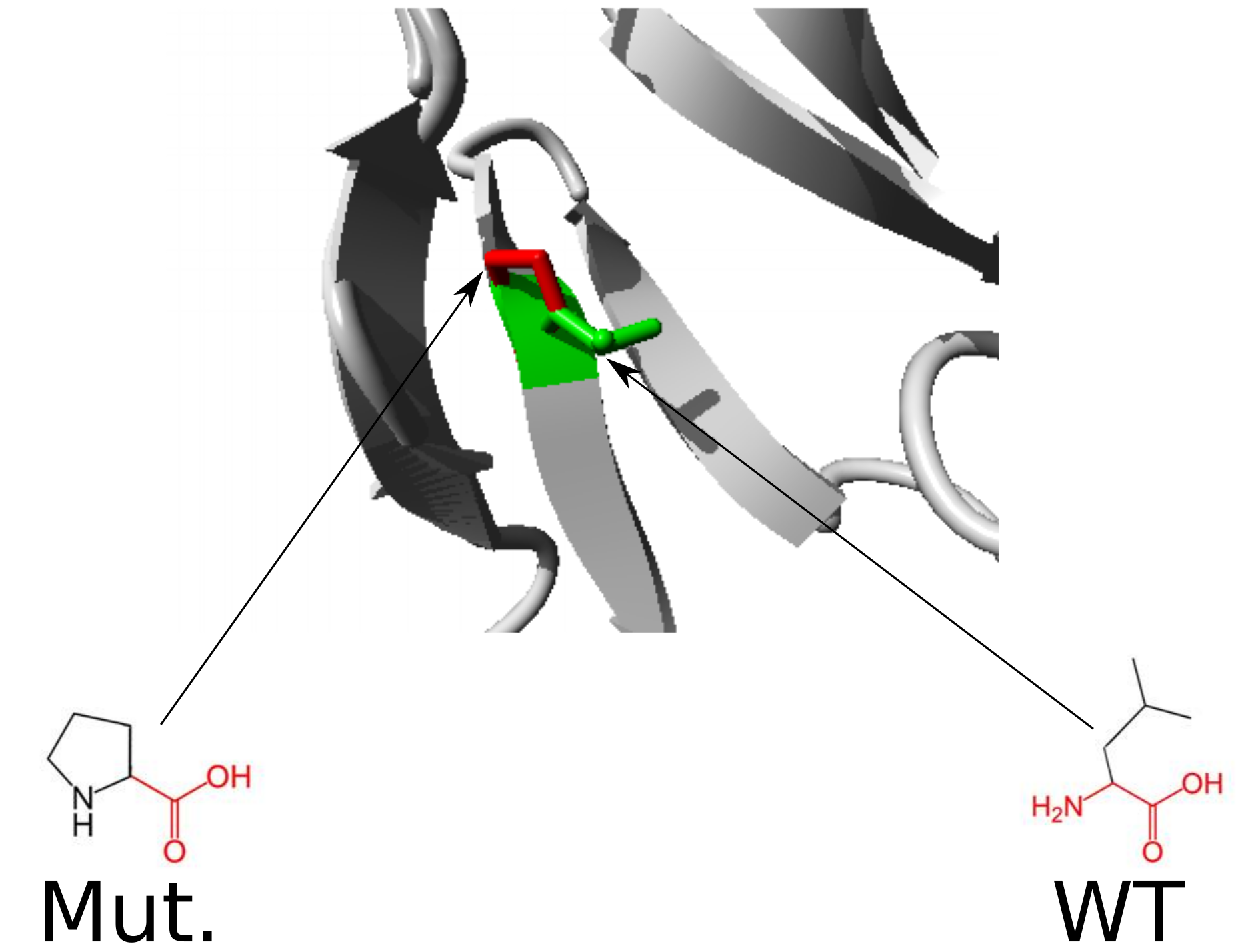


**Sup Fig. S7.** *CDH1* (NM_004360.4:c.641T>C:p.L214P). The mutant residue is smaller than the wild-type residue. The mutation is located within a domain, annotated in UniProt as: Cadherin 1. The mutation introduces an amino acid with different properties, which can disturb this domain and abolish its function. The mutation will cause an empty space in the core of the protein.

**Sup Fig. S8**


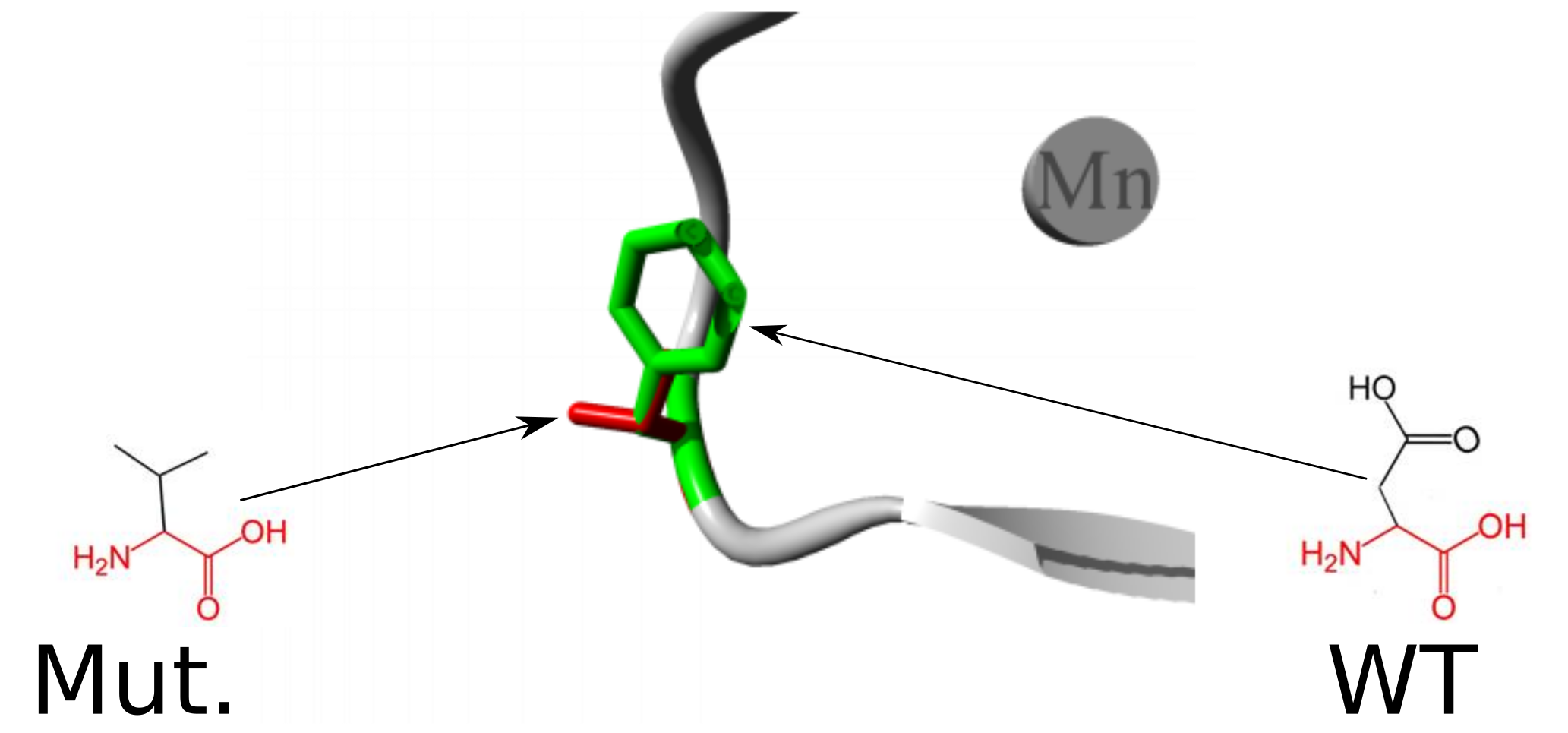


**Sup Fig. S8.** *CDH1* (NM_004360.4:c.1199A>T:p.D400V).The modified amino acid is smaller, has a neutral charge, and is more hydrophobic compared to the wild-type amino acid. Due to the loss of its negative charged wild-type amino acid, the protein may become less stable. The wild-type amino acid forms hydrogen bonds with Asp371, Ala408 and Asp458. Due to differences in size, the modified amino acid will not be able to form the same hydrogen bonds. This replacement is in the domain of Cadherin-3 and may interfere with its function. A conservative analysis shows only the wild-type amino acid, which indicates the pathogenicity of this substitution. This replacement may result in improper protein folding.

**Sup Fig. S9**


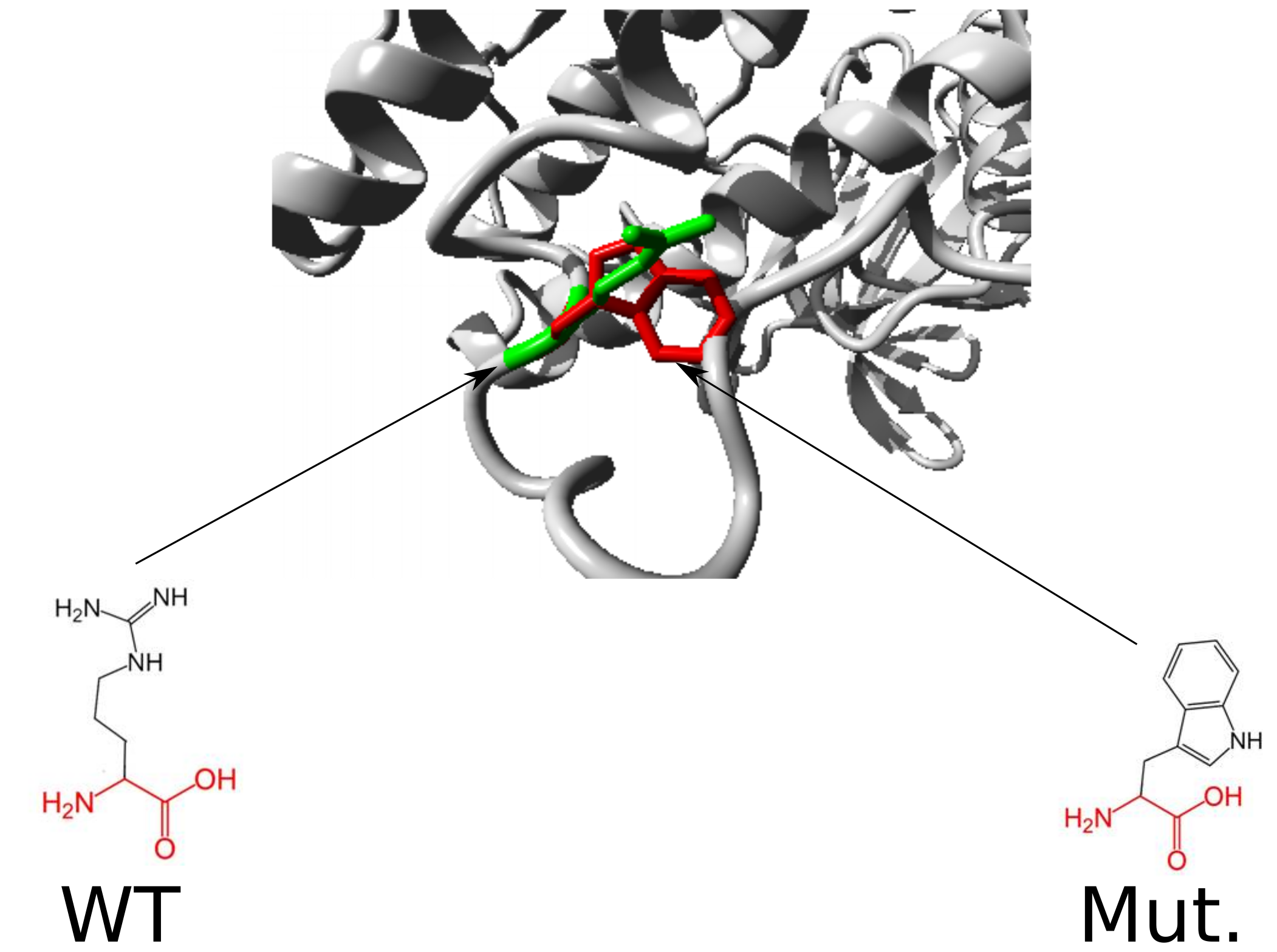


**Sup Fig. S9.** *STK11* (NM_000455: c.928C>T:p.R310W). The mutant residue is bigger than the wild-type residue. The wild-type residue charge was POSITIVE, the mutant residue charge is NEUTRAL.The mutant residue is more hydrophobic than the wild-type residue. The wild-type residue forms a hydrogen bond with: Alanine at position 318. The size difference between wild-type and mutant residue makes that the new residue is not in the correct position to make the same hydrogen bond as the original wild-type residue did. The difference in hydrophobicity will affect hydrogen bond formation. The wild-type residue forms a salt bridge with: Glutamic Acid at position 317. The difference in charge will disturb the ionic interaction made by the original, wild-type residue. The wild-type residue was buried in the core of the protein. The mutant residue is bigger and probably will not fit. The mutation will cause loss of hydrogen bonds in the core of the protein and as a result disturb correct folding.

**Sup Fig. S10**


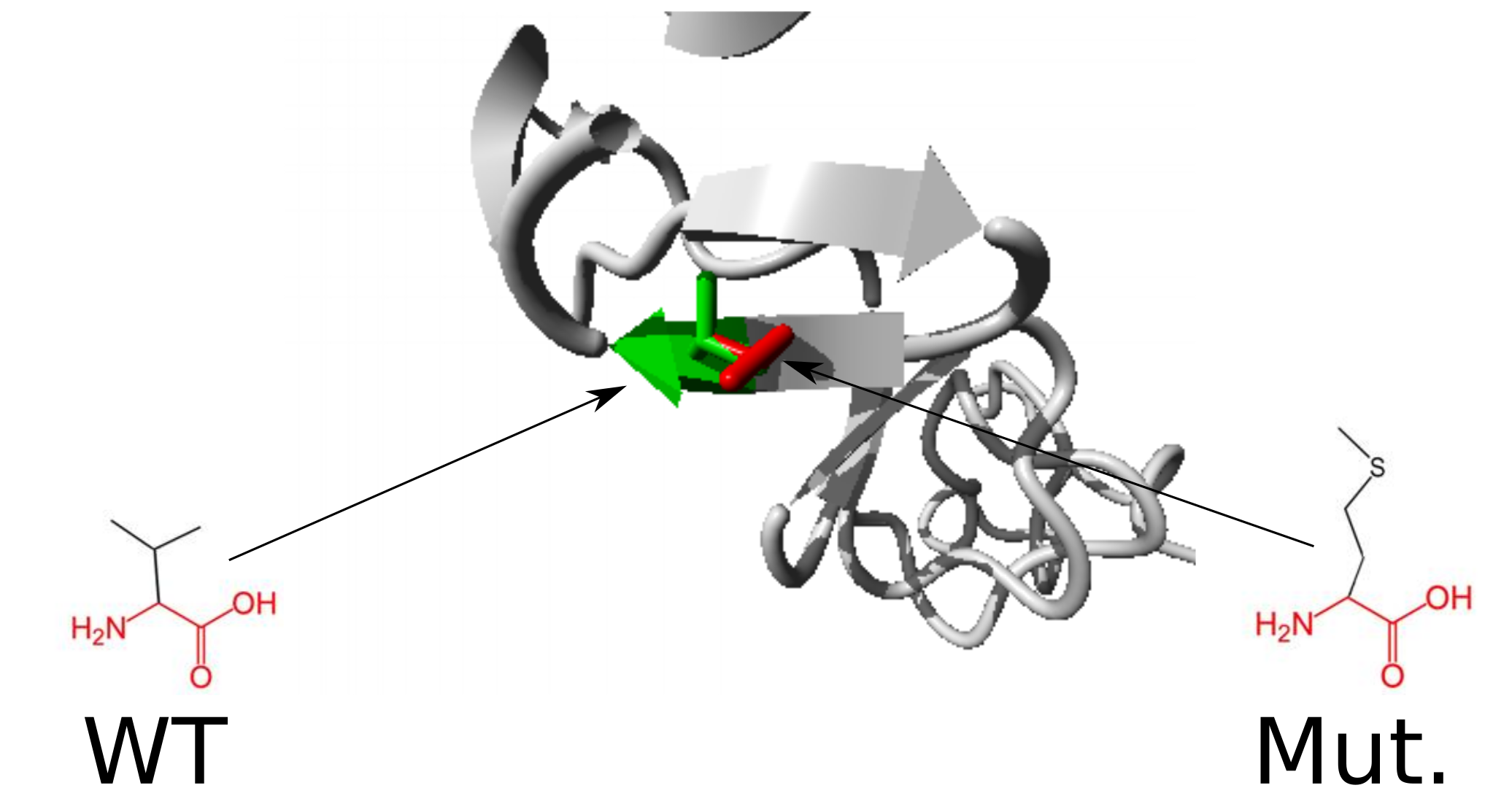


**Sup Fig. S10.** *EGFR* (NM_005228.3:c.874G>A:p.V292M). The mutant residue is bigger than the wild-type residue. The mutated residue is located very close to a residue that makes a cysteine bond. This cysteine bond itself is not mutated but could be affected by the mutation located in its vicinity. The mutation is located within a stretch of residues that is repeated in the protein, this repeat is named Approximate. The mutation into another residue might disturb this repeat and consequently any function this repeat might have. The wild-type residue is very conserved, but a few other residue types have been observed at this position too. Your mutant residue was not among the other residue types observed at this position in other, homologous proteins. However, residues that have some properties in common with your mutated residue were observed. This means that in some rare cases your mutation might occur without damaging the protein. The residue is located on the surface of the protein, mutation of this residue can disturb interactions with other molecules or other parts of the protein.
